# The associations between social determinants of health, mental health, substance-use and recidivism: a ten-year retrospective cohort analysis of women who completed the connections programme in Australia

**DOI:** 10.1186/s12954-023-00909-4

**Published:** 2024-01-03

**Authors:** Layla Maree Edwards, Sungwon Chang, Reem Zeki, Sacha Kendall Jamieson, Julia Bowman, Craig Cooper, Elizabeth Sullivan

**Affiliations:** 1 University of Technology Sydney, Sydney, Australia; 2 Justice Health and Forensic Mental Health Network, Malabar, Australia; 3 The University of Sydney, Sydney, Australia; 4 University of Newcastle Australia, Newcastle, Australia

**Keywords:** Women, Social determinants of health, Substance-use, Mental health, Recidivism

## Abstract

**Background:**

Women with substance-use issues are overrepresented in prison. Research on women’s recidivism often focuses on offending behaviour rather than the health and social circumstances women are experiencing when reimprisonment occurs. This study examines the relationship between social determinants of health (SDOH), mental health, substance-use and recidivism among women exiting prison with histories of substance-use.

**Methods:**

A retrospective cohort study of women exiting prison who completed the transitional support programme “Connections” between 2008 and 2018. Recidivism was measured up to two years post-release. Women’s support needs were measured at baseline (4 weeks pre-release) and follow-up (four weeks post-release). Ongoing needs in relation to well-established SDOH were calculated if: (1) at baseline women were identified as having a re-entry need with housing, employment, finances, education, domestic violence, child-custody and social support and (2) at follow-up women reported still needing help in that area. Women’s self-reported substance-use and mental health since release were captured at follow-up. Descriptive statistics were calculated for all measures. Associations between SDOH, mental health, substance-use and recidivism were estimated by multiple logistic regression, adjusting for potential confounders. We also evaluated the mediating effects of mental health on the relationship between SDOH and substance-use.

**Results:**

Substance-use was associated with increased odds of recidivism (adjusted odds ratio (AOR) 1.8, 95% confidence interval (CI) 1.1–2.9; *p* = 0.02). Poor mental health (AOR 2.9, 95% CI 1.9–4.6; *p* =  < 0.01), ongoing social support (AOR 3.0, 95% CI 1.9–5.0; *p* =  < 0.01), child-custody (AOR 1.9, 95% CI 1.0–3.3 *p* = 0.04), financial (AOR 2.0, 95% CI 1.3–3.2; *p* =  < 0.01) and housing (AOR 1.8, 95% CI 1.1–2.9; *p* = 0.02) needs were individually associated with increased odds of substance-use. Mediation analysis found mental health fully mediated the effects of ongoing housing (beta efficiency (*b*) = − 033, standard error (SE) 0.01; *p* = 0.05), financial (*b* = 0.15, SE 0.07; *p* = 0.05), child-custody (*b* = 0.18, SE 0.01; *p* = 0.05) and social support (*b* = 0.36, SE 0.1; *p* = 0.05) needs onto substance-use, and partially mediated the effects of domestic violence (*b* = 0.57, SE 0.23; *p* = 0.05) onto substance-use.

**Conclusion:**

This study underscores the critical importance of addressing the interplay between SDOH, mental health, substance-use and recidivism. An approach that targets SDOH holds the potential for reducing mental distress and substance-use, and related recidivism.

## Background

There are a number of important links between social determinants of health (SDOH) and the pathways of imprisonment for women. SDOH are the non-medical causes of health outcomes, encompassing the social, economic, political and environmental factors that impact the health of individuals and populations [1, 2]. SDOH include: adverse childhood experiences; education, income/monetary support, employment/job security and working life conditions; housing and neighbourhood conditions; social support/inclusion; socioeconomic position; and access to affordable health services [3–7]. Whilst these SDOH are recognised as determinants of health outcomes and health inequalities, many of these determinants have also been associated with women’s criminal offending [8, 9].

Women with a history of incarceration often have experiences of multiple disadvantages [10] including poverty and low income, unemployment and homelessness—all known SDOH [10–12]. Disproportionately high numbers of women in prison report histories of substance-use and diagnosis of mental illness [13–20]. The correlation between substance-use and criminal offending is well established [21] and for women, illicit drug charges is the most common serious offence among sentenced women in Australia and women in prison worldwide [22–24]. However, substance-use is not simply an individual choice or behaviour but a complex multifactorial health issue, correlated with SDOH [25]. For women in prison, substance-use is often shaped by physical and sexual victimisation across the life span resulting in cumulative trauma, subsequent poor mental health [13, 14, 26–33], as well as socioeconomic disadvantage [34, 35]. It is often reported by women in prison that experiences of trauma and mental illness precede substance-use, with women reportedly using substances to alleviate distress associated with trauma and to manage mental health issues [13, 14, 19]. Mental health and substance-use are therefore interrelated and complex health issues that not only increase a woman’s risk of imprisonment [36, 37] but may also impact women’s integration into the community post-release, and therefore reimprisonment.

In 2019, 9535 women were either on remand or sentenced to a correctional facility in Australia with approximately the same number of women (*n* = 9573) released to the community [38]. The rate of women who return to prison following a release from prison (i.e., recidivism) is not routinely published in Australia [39, 40]. However, of all people (both men and women) released from prison in Australia in 2019*,* 42.7% returned to prison within two years (returning 2021–2022) [40]. Whilst recidivism rates are not reported for women, the proportion of women in prison who have a prior imprisonment is reported which shows that roughly half (48%) of women in prison have been previously imprisoned [11]. New South Wales (NSW), the most populous State in Australia, housed 28% (or *n* = 1014) of Australia’s female prison population in March 2020 [41]. Of which, 72% reported a previous imprisonment [42]. This data not only shows the high volume of reincarceration of women, but also the reality that surviving in the community after release from prison is difficult for women with a history of incarceration, many of whom have co-occurring substance-use and mental health issues.

Whilst it is important to report rates of recidivism, the use of this measure to evaluate or measure the effectiveness of interventions has it flaws. Using a simplified measure of recidivism with an emphasis on individual choice and behaviour decontextualizes the complex systemic issues impacting women’s access to resources and support after release from prison [12, 36]. In this way, a measure like recidivism obfuscates how survival in the community post-release is shaped by broader social and political forces (i.e., SDOH) that deprive individuals of choice and the means to pursue long-term change in their life [12]. However, research with incarcerated women rarely investigates key SDOH within their exploration of recidivism.

A recent systematic review [36] looking at the effectiveness of post-release or transitional (i.e., via a throughcare approach) programmes for women with substance-use disorders, reported that neither SDOH nor mental health were reviewed as associating factors in individual studies. Effectiveness of programmes was measured by recidivism (yes/no) in all included studies (*n* = 12) and only four (33%) reported substance-use uptake post-release despite the study population for all studies being women with substance-use disorders. Housing, employment, finances, education, child-custody, social support nor mental health were analysed, despite their influence on health and their associations with imprisonment [12, 43].

When it comes to “what works” to reduce women’s reoffending, there remains a lack of evidence as to what is effective at reducing recidivism for women exiting prison with histories of substance-use. This highlights a need to look beyond rates of recidivism to glean how SDOH and complex health issues can impact community integration for women exiting prison. Such research will shed light on the particular challenges women are facing when they re-enter the community and advance understandings of the pathways to reimprisonment among women. Therefore, this study aims to investigate the relationship between SDOH, substance-use, mental health and recidivism among women, identifying factors associated with the reimprisonment of women with histories of substance-use.

## Methods

### Study design and participant selection

This is a retrospective cohort study of women who completed the Connections programme (referred to as Connections) between 1st January 2008 and 30th June 2018, allowing for a two-year observation period for recidivism outcomes. The source of the study cohort is the Connections database, which is maintained by the Justice Health and Forensic Mental Health Network. Eligibility criteria for Connections is reported elsewhere [44]. To be selected for this study, we included all women who completed Connections (i.e., completed pre- and post-release assessments) for the first time. Women were allocated to one of the following groups based on their recidivism status at two years post-release: (1) return-to-custody (RTC) group or (2) did not return-to-custody (No-RTC) group.

### The Connections programme

Connections is a voluntary prison health programme for people in prison with histories of illicit substance-use available in all adult correctional facilities in NSW, Australia. Full details of Connections can be found in the published programme evaluation study protocol [44]. Briefly, Connections clinical support workers (CSWs) meet face-to-face with people in prison who are preparing to exit to the community. Given that Connections is a health programme, CSWs refer to participants as patients. CSWs engage with patients generally up to four weeks prior to release to provide pre-release assessment and complete a re-entry treatment plan. This forms the foundation for linkage to community-based support and services related to the individual needs of each patient, with their direct input.

After release, CSWs continue to provide support up to four weeks post-release (with an option to extend for another four weeks, if requested). Due to the individualised nature of this programme, there is no set number of engagements between CSWs and patients. At four weeks, CSWs administer the post-release assessment to track progress made in the community and to identify if patients require further support for any ongoing or new needs. Patients who requested extended support did not complete any further assessments. For this reason, the proportion of patients who requested additional support are not reported.

## Measures

### Social determinants of health

Housing, employment, financial problems, education, domestic violence, parenting and social support were categorised as SDOH as they represent fundamental areas that can impact a wide range of health outcomes [45–49]. The re-entry treatment plan outlines whether patients had any “issues identified” or “no issues identified” in the following areas: housing, employment/training, financial, education, domestic violence, parenting and social support. Ongoing needs with SDOH were calculated when patients were identified as (1) having an *issue identified* at baseline (via the pre-release assessment) and (2) then at follow-up (via the post-release assessment) reported they still *need help* or have *problems* in that same area. Analysing ongoing needs allows us to review changes in need over time, highlighting areas that need targeted resources. Women who had *no issue identified* at baseline, or women who were identified as having an issue at baseline but at follow-up no longer had that need/problem, were allocated to the comparison group. Women who did not answer follow-up questions were also allocated to the comparison group to ensure we reported on the true number of women who self-reported needing help compared those who did not report needing help. See Table A1 in the appendix for a detailed description of all the variables tested in the analyses.

### Complex health issues

Substance-use and mental health are categorised as complex health issues which can be influenced by SDOH; are associated with imprisonment; and women with these health issues are overrepresented in prisons [9, 36, 50, 51]. Substance-use issues were identified at baseline by the re-entry treatment plan. Mental health was measured using the mental health dimension of the Short Form-12 Health Survey (SF-12).

The SF-12, which was derived from the 36-Item Short Form Health Survey (SF-36), is a health-related quality-of-life instrument that has been demonstrated to be a reliable and valid use of self-reported health status among general and specific populations [52–54]. Large population-based studies have found the mental health component of the SF-12 to be a useful screening tool for monitoring the prevalence of mental health issues (including depression, major depression, bipolar disorder and anxiety disorder) among general populations and for targeting treatment and prevention [55, 56]. It uses scored scales based upon the US general population, with a mean of 50 and standard deviation of 10. A score below 50 indicates poorer mental health than the average population [52]. The mean threshold of < 50 (10) has been found to be an appropriate measure of poor mental health among Australian populations [56, 57] and to be a psychometrically sound instrument for measuring mental health among people with severe mental illness (including schizophrenia, schizoaffective, bipolar and major depression) [58]. The proportion of women who had *issues identified* on the re-entry treatment plan, or women who score < 50 on the SF-12, were reported and used for baseline data.

At follow-up, the post-release assessment asked questions about these health issues since release: substance-use was identified when women self-reported either (1) using any drugs (other than prescribed) and/or (2) having had problems with alcohol since release from prison; mental health was measured using the SF-12. Unlike SDOH which reflect an ongoing need in those areas, our analysis of health conditions included all women who reported substance-use or poor mental health since release from prison (i.e., not ongoing). This was done to account for the challenges women specifically face when exiting prison which might trigger adverse mental health or substance-use after release from prison [59–61]. Women who did not report substance-use at follow-up, and women who scored > 50 on the SF-12 were allocated to the comparison group. Women who did not answer the relevant follow-up questions were also allocated to the comparison group.

### Statistical analysis

All data analysis was performed using IBM Statistical Package for the Social Sciences (SPSS) (version 27) software [62]. Frequencies for categorical variables and means with standard deviations for continuous variables were calculated. Statistical differences between RTC group and no-RTC group women during a two-year follow-up were determined with Chi-square tests and independent-sample t tests as appropriate. Statistical significance was defined at the *p* value < 0.05.

Two backward elimination, also called backward stepwise multiple logistic regression, models were used, one with the predictor variable recidivism (i.e., being allocated to the RTC group) and the second with the predictor variable substance-use since release from prison. Backward elimination begins with all of the variables of interest in the equation (i.e., SDOH and complex health issues) and removes the least significant variables one-by-one until all remaining variables are statistically significant. The first multiple logistic analysis was used to assess the associations between SDOH, complex health issues (independent variables) with being allocated to the RTC group (dependant variable). In this model, we adjusted for admission year to account for programme changes and improvements between 2008 and 2018. In addition, we adjusted for age, being from a culturally and linguistically diverse background, Aboriginal and Torres Strait Islander status, first time in custody and sentence-length as the literature has reported these factors to be correlated to recidivism [50, 51, 63].

Substance-use is often cited as a criminogenic factor [36, 50]; however, there are gaps in knowledge that explore factors associated with substance-use after release from prison. To address this gap, we used a second multiple logistic regression model to assess the associations between SDOH and mental health (independent variables) with women who reported substance-use since release from prison (dependant variable) to explore potential influential factors driving women to return to substance-use in the community. The second model was adjusted for admission year (to account for programmes changes), age (as age (younger) has been found to be a predictor of substance-relapse [64]), and Opioid Substitution Therapy status in prison (to account that some women were on treatment which has shown reductions in post-release relapse [65]). Crude and adjusted odds ratios (AORs) and 95% confidence intervals (CIs) were produced.

**Fig. 1:**
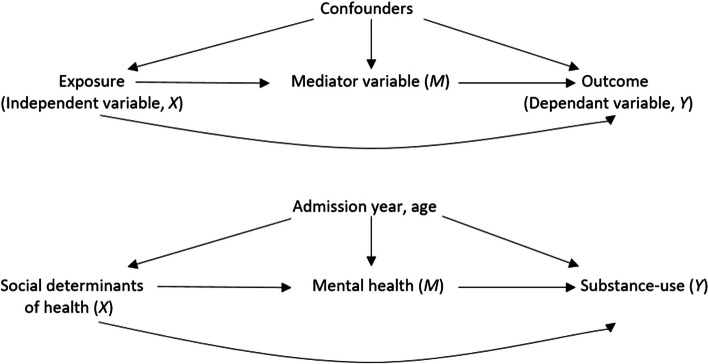
Directed acyclic graph (DAG)

Finally, considering women in prison have reported that mental distress often preceded substance-use [66–68], and previous studies have found that complex health issues can be directly influenced by SDOH [5]; we hypothesised that mental health (*M*) will mediate the effect of SDOH (*X*) onto substance-use (*Y*) (See Fig. 1). To test this theory, we performed a mediation analysis using the PROCESS macro (model 4, version 4.2) by Andrew Hayes [69] in SPSS, adjusting for admission year and age. Beta coefficients (b) and standard error (SE) were computed. Statistical significance of the indirect effect was determined using 10,000 bootstrap confidence intervals, as these have higher power to detect a statistically significant indirect effect [70].

### Ethics

Ethics approval for this project was granted from NSW Population and Health Services Human Research Ethics Committee (HREC) (HREC/16/CIPHS/17); Aboriginal Health Medical Research Council HREC (1187–16); Justice Health and Forensic Mental Health Network HREC (Reference number: G217-16); Corrective Services NSW Ethics Committee (D190221581); University of Technology Sydney (ETH18-2587); and the University of Newcastle (H-2020–0074).

## Results

A total of *n* = 413 (4.0%) women, who had their first engagement with Connections and completed both assessments (pre- and post-release assessments), were included in this study. Of those 40.2% (*n* = 166) were allocated to the RTC group and 59.8% (*n* = 238) were allocated to the no-RTC group. Sociodemographic characteristics by group are shown in Table 1. RTC group women were significantly younger (mean age of 32.8 (SD = 7.3) vs. 34.8 (SD = 8.1; *p* = 0.05)); larger proportions identified as Aboriginal or Torres Strait Islander (41.0% vs. 26.5%; *p* = 0.04); had lower educational attainment (5.6% vs. 19.6%; *p* = 0.01); and fewer had participated in previous formal employment (33.1% vs. 21.7%; *p* = 0.01) compared to no-RTC group women. Most women (76.8%) reported having children. When asked about histories of substance-use, a larger proportion RTC group women reported a drug problem prior to incarceration (89.5% vs. 79.5%; *p* = 0.01) compared to no-RTC group women. Whilst no differences were seen between groups, most women reported previously receiving treatment for a mental health condition (71.6% vs. 74.0%; *p* = 0.60).

**Table 1:** Sociodemographic characteristics by group

Sociodemographic demographics	Total *n* (%)^a^ *n* = 413	Women *n* (%)^a^		*p-*value
		Return-to-custody within two years (RTC) group *n* = 166 (40.2%)	Did not return-to-custody within two years (No-RTC) group *n* = 238 (59.8%)	
Mean age (SD)	34.0 (7.8)	32.8 (7.3)	34.8 (8.1)	**0.05**
*Country of birth*				
Australia	361 (92.1)	147 (91.9)	214 (92.2)	0.89
Overseas	31 (7.8)	11 (7.3)	20 (8.0)	
*Aboriginal and Torres Strait Islander Status*				
Indigenous	118 (32.8)	64 (41.0)	54 (26.5)	**0.04**
Non-Indigenous	242 (67.2)	92 (59.0)	150 (73.5)	
*Marital status*				
Never Married	234 (60.3)	100 (62.1)	134 (59.0)	0.69
Married/Defacto/Regular partner	112 (28.9)	46 (28.6)	66 (29.1)	
Divorced/Separated/Widowed	42 (10.8)	15 (9.3)	27 (11.9)	
*Education attainment*				
No school certificate (did not complete high school)	340 (86.1)	152 (94.4)	188 (80.3)	** < 0.01**
High School Certificate (completed high school)	31 (7.8)	5 (3.1)	26 (11.1)	
Tertiary education (post high school qualified)	24 (6.1)	4 (2.5)	20 (8.5)	
*Employment history*				
Have been previously employed	286 (73.5)	109 (66.9)	177 (78.3)	**0.01**
Never previously employed	1036 (26.5)	54 (33.1)	49 (21.7)	
*Parent status*				
Yes	298 (76.8)	127 (78.9)	171 (75.3)	0.41
No	90 (22.3)	34 (21.1)	56 (24.7)	
*Drug problem (prior to current incarceration)*				
Yes	327 (83.6)	145 (89.5)	182 (79.5)	**0.01**
No	64 (16.4)	17 (10.5)	47 (20.5)	
*Prior to this incarceration, women received treatment by a psychiatrist/doctor or Community Mental Health team for a mental health problem?*				
Yes	284 (73.0)	116 (71.6)	168 (74.0)	0.60
No	105 (27.0)	46 (28.4)	59 (26.0)	

^a^Those who did not answer or refused to answer were removed from analysis; as a result, total numbers do not equal total number of women but total number of people who answered each question

^b^ Nine women’s’ RTC status was missing; as a result, the group numbers do not equal the total number of women included

### Incarceration history by group

Table 2 presents the incarceration history by group. Significantly more RTC group women had a history of imprisonment (80.4% vs. 67.7%; *p* = 0.01) and were imprisoned for substance-related offences (79.8% vs. 67.4%; *p* = 0.01) compared to no-RTC group women. No significant differences were seen between groups for sentence-length and probation after release.

**Table 2:** Incarceration history by group

Incarceration history	Total *n* (%) ^a^ *n* = 413	Women *n* (%) ^b^		*p-*value
		Return-to-custody within two years (RTC) group *n* = 166 (40.2%)	Did not return-to-custody within two years (No-RTC) group *n* = 238 (59.8%)	
*First time in custody*				
Yes	106 (27.0)	32 (19.6)	74 (32.3)	**0.01**
No	307 (75.2)	131 (80.4)	155 (67.7)	
*Imprisoned for substance-related offences*				
Yes	283 (72.6)	130 (79.8)	153 (67.4)	**0.01**
No	1070 (27.4)	33 (20.2)	74 (32.6)	
*Length of current sentence*				
< 6 months	148 (38.4)	58 (36.0)	90 (40.2)	0.43
6—< 12 months	142 (36.9)	58 (36.0)	84 (37.5)	
1 year or more	95 (24.7)	45 (28.0)	50 (22.3)	
*On probation after release*				
Yes	302 (78.4)	129 (79.6)	173 (77.6)	0.63
No	83 (21.6)	33 (20.4)	50 (22.4)	

^a^Those who did not answer or refused to answer were removed from analysis; as a result, total numbers do not equal total number of women but total number of women who answered each question

^b^Nine women’s’ RTC status was missing; as a result, the group numbers do not equal the total number of women included

### Issues at baseline and needs at follow-up by group

Table 3 shows the proportion of women who were identified as having an issue at baseline and their needs at follow-up. Overall women had a mean of 6.3 (SD = 2.2) issues at baseline, with a mean of 5.0 (SD = 1.9) SDOH issues and 1.3 (SD = 0.6) complex health issues per woman at baseline. At follow-up, women reported a mean of 2.1 (SD = 1.7) total needs per woman, with a mean of 1.3 (SD = 1.2) SDOH ongoing needs and 0.8 health issues per woman at follow-up. Nearly all women had a substance-use issue at baseline (93.7%). At follow-up, a larger proportion RTC group women reported substance-use since release (38% vs. 28.2%; *p* = 0.04) compared to no-RTC group women.

**Table 3:** Issue identified at baseline and needs at follow-up by group

Needs	Issues identified at baseline *n* = 413 (%)^a^	Needs at follow-up^b,c^ *n* = 404	Women *n* (%)		*p-*value
			Return-to-custody within two years (RTC) group *n* = 166 (40.2%)	Did not return-to-custody within two years (No-RTC) group *n* = 238 (59.8%)	
*Social Determinants of Health*					
Total SDOH, mean (SD)	5.0 (1.9)	1.3 (1.2)	1.3 (1.3)	1.3 (1.2)	0.27
Housing	343 (83.1)	105 (26.0)	48 (28.9)	57 (23.9)	0.26
Employment	332 (80.4)	26 (6.4)	11 (6.6)	15 (6.3)	0.90
Financial problems	383 (92.7)	196 (48.5)	79 (47.6)	117 (49.2)	0.76
Education	314 (76.0)	21 (5.2)	9 (5.4)	12 (5.0)	0.90
Domestic violence	86 (20.8)	14 (3.5)	4 (2.4)	10 (4.2)	0.33
Child-custody	250 (81.7)	62 (20.8)	30 (23.6)	32 (18.7)	0.31
Social support	285 (69.0)	98 (24.3)	42 (25.3)	56 (23.5)	0.68
*Complex* *health* *issues*					
Total health issues, mean (SD)	1.3 (0.6)	0.8 (0.8)	0.8 (0.8)	0.7 (0.7)	0.11
Substance-use	387 (93.7)	130 (32.2)	63 (38.0)	67 (28.2)	**0.04**
Mental health	164 (39.7)	188 (46.5)	77 (46.4)	111 (46.6)	0.96
*Total needs*					
Total needs, mean (SD) ^d^	6.3 (2.2)	2.1 (1.7)	2.2 (1.8)	2.0 (1.6)	0.16

^a^SDOH = Social determinants of health

^b^Social determinants of health are defined as ongoing needs. They are the proportion of women who had an “issue” at baseline and reported still having that need at follow-up, whereas complex health issues were analysed at follow-up only (i.e., post-release), and therefore are not categorised as ongoing

^c^Nine women’s’ RTC status was missing, and therefore, we excluded them from the analysis

^d^Mean total needs are the mean SDOH and health issues per woman

### Multiple logistic analysis

The adjusted multiple logistic regression showed that substance-use after release was associated with an increase in odds of RTC within 2 years (AOR 1.8, 95% CI 1.1–2.9; *p* = 0.02) compared to women who did not report substance-use during follow-up (Table 4). No other SDOH or health issue had a direct relationship with recidivism.

**Table 4:** Odds ratio of correlates of return-to-custody within two years, ongoing social determinants of health and complex health issues at follow-up

Needs at follow-up	Adjusted odds ratio^a^	(95% CI)	*p-*value
*Social determinants of health*^*b*^			
Housing	1.1	(0.6–1.9)	0.75
Employment	0.9	(0.3–2.1)	0.73
Financial problems	1.0	(0.6–1.6)	0.99
Education	1.1	(0.2–8.0)	0.93
Domestic violence	0.5	(0.1–2.0)	0.31
Child-custody	1.3	(0.7–2.3)	0.44
Social support	1.3	(0.8–2.2)	0.31
*Complex health issues*^*c*^			
Substance-use	**1.8**	**(1.1–2.9)**	**0.02**
Mental health	1.1	(0.7–1.9)	0.6

^a^Adjusted for admission year, age, born overseas, Aboriginal and Torres Strait Islander status, first time in custody and sentence-length

^b^Social determinants of health are defined as ongoing needs. They are the proportion of women who had an “issue” at baseline and reported still having that need at follow-up

^c^Complex health issues were analysed at follow-up only (i.e., post-release), and therefore are not categorised as ongoing

As the association between criminal offending and substance-use is well established, we wanted to explore whether any SDOH or mental health were individually associated with substance-use (Table 5). After adjusting for potential confounders, having poor mental health at follow-up (AOR 2.9, 95% CI 1.9–4.9; *p* =  < 0.01), or reporting ongoing needs with social support (AOR 3.0, 95% CI 1.9–5.0; *p* =  < 0.01), child-custody (AOR 1.9, 95% CI 1.0–3.3 *p* = 0.04), finances (AOR 2.0, 95% CI 1.3–3.2; *p* =  < 0.01) and housing (AOR 1.8, 95% CI 1.1–2.9; *p* = 0.02) were associated with increased odds of substance-use post-release.

**Table 5:** Odds ratio of correlates of social determinants of health, mental health and substance-use

Needs at follow-up ^a^	Adjusted odds ratio ^b^	(95% CI)	*p-*value
*Social determinants of health *^*c*^			
Housing	**1.8**	**(1.1–2.9)**	**0.02**
Employment	0.9	(0.4–2.2)	0.79
Financial problems	**2.0**	**(1.3–3.2)**	** < 0.01**
Education	1.0	(0.4–2.6)	0.99
Child-custody	**1.9**	**(1.0–3.3)**	**0.04**
Social support	**3.0**	**(1.9–5.0)**	** < 0.01**
*Complex health issues *^*d*^			
Mental health	**2.9**	**(1.9–4.6)**	** < 0.01**

^a^ Domestic violence was removed from the analysis as the model was unstable due to the small number of women reporting these needs

^b^Adjusted for admission year, age, and Opioid Substitution Therapy status in prison

^c^ Social determinants of health are defined as ongoing needs. They are the proportion of women who had an “issue” at baseline and reported still having that need at follow-up

^d^ Complex health issues were analysed at follow-up only (i.e., post-release) and therefore are not categorised as ongoing

### Mediators of treatment effects

**Fig. 2:**
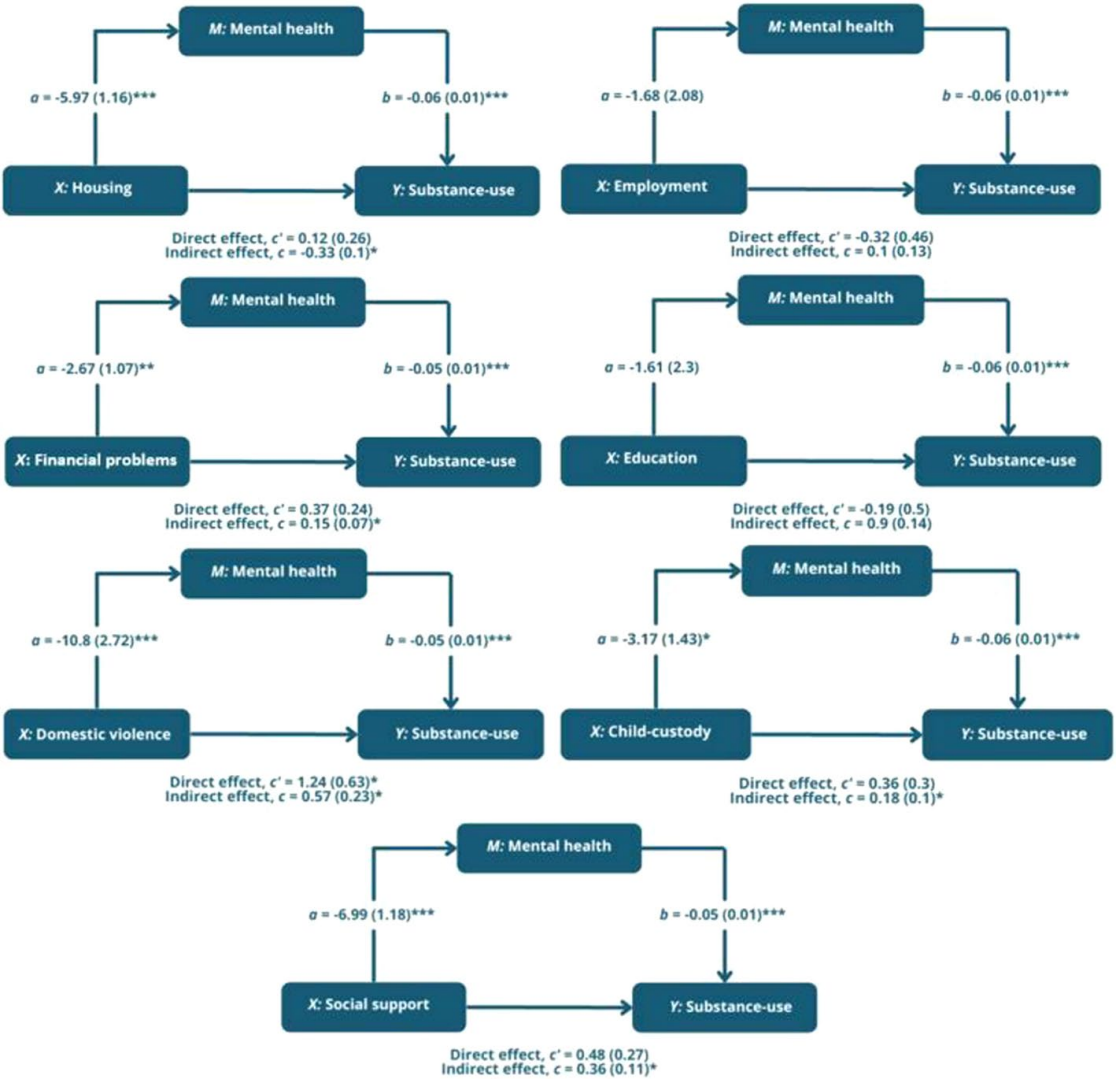
Indirect effects of social determinants of health onto substance-use through mental health in mediation model. *Note*: *p = 0.05; **p = 0.01; ***p =  < 0.01

The mediation models assessed the mediating role of mental health on the relationship between SDOH and substance-use. The results showed poor mental health to have a significant indirect effect on the relationship between ongoing housing (*b* = − 0.33, SE 0.01), financial (*b* = 0.15, SE 0.07), domestic violence (*b* = 0.57, SE 0.23), child-custody (*b* = 0.18, SE 0.01) and social support (*b* = 0.36, SE 0.1) needs and substance-use, supporting our hypothesis (see Fig. 2 and Table A2 in the appendix). Furthermore, the direct effect (path *c’*) of domestic violence on substance-use in the presence of the mediator was also significant (*b* = 1.24, SE 0.63, *p* = 0.05). Hence, mental health partially mediated the relationship between domestic violence and substance-use. In all other significant models, mental health fully mediated the relationship between ongoing housing, financial, child-custody and social support needs and substance-use, as the direct effect (path *c’*) was not significant in the presence of mental health.

## Discussion

The relationship between SDOH, mental health, substance-use and recidivism was examined in a population of women who participated in Connections for the first time between 2008 and 2018. RTC group women were significantly younger; with higher proportions identifying as Aboriginal or Torres Strait Islander, having lower educational attainment and limited work experience compared to no-RTC group women. These findings are consistent with existing research that has found younger age, low educational attainment, unemployment and being a First Nations person or person from a culturally and linguistically diverse community to be correlated with recidivism [71–75].

### Pathways to reimprisonment

Larger proportions of RTC group women reported a history of drug use (prior to imprisonment), were currently incarcerated for substance-related offences and reported substance-use (either using drugs (other than prescribed) or having a problem with alcohol) since release from prison compared to no-RTC group women. In addition, substance-use since release from prison increased the odds of recidivism by 80% compared to women who did report substance-use since release. These findings not only confirm the association between substance-use and pathways to imprisonment reported elsewhere [16, 21, 23, 76], but also indicate that substance-use after release is a pathway to reimprisonment, specifically for women.

### Pathways to substance-use post-release

Whilst predictors of recidivism have gained attention due to the increasing number of people who RTC after release, to-date no paper has examined associations between SDOH, mental health and substance-use after release from prison among women. As a result, we sought to understand if any SDOH or mental health were associated with substance-use post-release. Our results illustrate that poor mental health in tandem with ongoing social support, child-custody, financial and housing needs were individually associated with substance-use post-release.

Substance-use was increased by nearly threefold among women who had a mean score of < 50 on the SF-12, indicating poor mental health; by threefold for women who reported ongoing social support needs (i.e., negative social support); by twofold for women who reported ongoing financial problems; and the odds of substance-use were increased by 80% among women who reported ongoing child-custody (85%) and housing (81%) needs.

Whilst these SDOH increased the odds of substance-use, it is potentially through the understandable worry and distress these issues cause. Furthermore, as substance-use can be influenced by a variety of causes, it is unrealistic to assume that a single variable would explain a causal relationship. Although our data cannot test whether poor mental health came prior to substance-use, other research recognises poor mental health as a common pathway to substance-use uptake [66, 68, 77, 78]. These studies highlighted that not only did mental distress precede the onset of substance-use but long-term substance-use (as a form as self-medication) led to substance dependency, a known pathway to imprisonment [21, 79, 80]. In addition, literature reviewing ways to improve/support abstinence shows that negative (or inadequate) social support [81], issues regaining custody of children [82] and child removal [83–85], financial stress [86–89] and housing insecurities [90, 91] can increase the risk of substance-use via triggering mental distress.

Finally, the mediation models found that ongoing housing, financial, child-custody and social support needs were associated with substance-use only via poor mental health when it was added as a mediating variable. Domestic violence, however, had partial indirect effects onto substance-use through mental health, as the indirect effect did not cancel out the direct effect (path c’) when the mediator was introduced. Therefore, we argue that substance-use should be understood and treated in conjunction with co-occurring mental health in this population.

Previous literature has highlighted how broader structural barriers impact women’s transition to the community in regard to: the real challenges women face trying to obtain appropriate housing as a result of cost/affordability and the sheer paucity of government-funded housing [92–95]; gendered barriers to gaining meaningful employment and therefore reduced opportunities for financial independence [96–99]; the catch-22 of trying to regain child-custody after a period of imprisonment but needing appropriate housing and a stable job that suits the needs of children to show stability [98, 100]; and how custody length, along with co-occurring substance-use and mental health, and socioeconomic disadvantage can further impact these ongoing needs for women who are exiting prison to the community [101, 102].

Therefore, unless changes are made at a social–political–environmental level to address these broader structural barriers (i.e., upstream SDOH), then prison and post-release policies and programmes are limited in how much they can support women with co-occurring substance-use and mental health issues, particularly in terms of achieving long-term sustainable outcomes. Moreover, with consideration of SDOH and a harm prevention approach to (re)incarceration, what is required is targeted resourcing of women’s services providing holistic wraparound support for women to improve access to appropriate and long-term housing, income support/employment opportunities, restoration of children, and domestic and family violence. Such support could see reductions in poor mental health and therefore reduced rates of substance-use post-release.

### Strengths and limitations

Research including incarcerated women has focussed too heavily on recidivism and individual behaviour, rather than on the broader social determinants of reimprisonment. This study extended our knowledge of the relationship between SDOH, mental health, substance-use and recidivism among women with histories of substance-use in a large Australian sample over ten years. These results are of value to decision makers in relation to policies and programmes that wish to improve community integration and reduce recidivism among women. As Connections focused on patients with substance-use histories, our findings may not be generalizable to the wider women’s prison population. However, considering most women in prison report a history of substance-use and poor mental health, the results of this study can potentially be applied to a larger number of women in prison in Australia and worldwide.

A number of study limitations should be considered when interpreting these results. Self-reported data is known to have its biases [103]. Higher proportions of women identified SDOH issues at baseline compared to still having that need at follow-up. We cannot reasonably expect that most patients needs have been met during the eight week support provided by Connections. However, given the aims of Connections is to provide linkage to community-based services, we can postulate that most of the needs would have been addressed (and linked) by CSW. In addition, we also suggest that context (78% of women were on probation after release), increased agency and sensitivity of the nature of many follow-up questions (i.e., housing status in the community, domestic violence, substance-use, etc.) may have reduced the response rate and impacted findings from reporting bias due to stigmatisation around substance-use, and or fear it might jeopardise their survival, reunification of their children, or government benefits and council housing applications/policies [104–109].

Recidivism is one of the most fundamental measures used in criminal justice research [110–113]. However, by grouping women based on their RTC status at two years post-release we decontextualize a complex series of events that explains why women returned to custody. Future prospective research in this area should endeavour to use a suite of recidivism events such as survival time in the community, offence type and severity. Such a suite of measures could give readers and policy makers a clearer view of the post-release experience and challenges women face after release from prison. The pre- and post-release assessments were designed as general health and wellbeing assessments for people in prison with histories of substance-use to identify the individual needs of each patient. They were not designed to capture the specific needs of women or people from different cultural groups. We acknowledge that colonisation and its ongoing impacts shape social, political and cultural determinants of First Nations people’s health and that neither this context, nor First Nations people’s health frameworks, have been captured by these assessments.

## Conclusion

Results from this study highlight that women who use substances after release from prison are more likely to return-to-custody. Substance-use post-release was seen to be influenced by SDOH, in the context of mental distress in some cases. Therefore, any policy or programme that aims to reduce recidivism for women must address social determinants of co-occurring substance-use and mental health. This requires political commitment to ensure services and programmes are resourced to provide holistic support for women exiting prison, as well as resourcing across the broader social welfare system to address housing affordability and funding for women’s services. Such a commitment could see improved wellbeing and reductions in substance-use following release and therefore recidivism.

## Data Availability

Not applicable.

## Appendix

See Tables

**Table 6:** Social determinants of health and complex health variables—data dictionary

Needs	Inclusion criteria	
	At baseline	Follow-up question
*Social determinants of health*		
Housing	Housing issues identified on the re-entry treatment plan *Yes, issues identified*—included in the analysis *No issues identified*—allocated to the comparison group	*Do you need help finding somewhere else to live?* *Yes*—Ongoing housing needs identified *No*—No ongoing housing need, allocated to the comparison group Did not answer question (9.0%)—allocated to the comparison group
Employment	Employment/training issues identified on the re-entry treatment plan *Yes, issues identified*—included in the analysis *No issues identified*—allocated to the comparison group	*Do you need any further help with finding employment or accessing training/education?* *Yes*—Ongoing employment needs identified *No*—No ongoing employment need, allocated to the comparison group Did not answer question (13.3%)—allocated to the comparison group
Financial problems	Financial issues identified on the re-entry treatment plan *Yes, issues identified*—included in the analysis *No issues identified*—allocated to the comparison group	*Do you have any money problems with the following: Centrelink (i.e., government support payment); Department of Housing; Bank loan; Bank account/Credit-card; Personal loan; Rental; Damage done to property; State Debt Recovery Office; Victims Compensation; Child support agency; Tax outstanding; Utilities (Electricity/phone); or Bankruptcy?* *Yes* (to any)—Ongoing financial problem identified *No*—No ongoing financial problem, allocated to the comparison group Did not answer question (9.4%)—allocated to the comparison group
Education	Educational issues identified on the re-entry treatment plan *Yes, issues identified*—included in the analysis *No issues identified*—allocated to the comparison group	*Do you need any further help with finding employment or accessing training/education?* *Yes*—Ongoing employment need identified *No*—No ongoing education need, allocated to the comparison group Did not answer question (13.3%)—allocated to the comparison group
Domestic violence	Domestic violence issues identified on the domestic violence screening sheet in the pre-release assessment *Yes, issues identified*—included in the analysis *No issues identified*—allocated to the comparison group	*How often in the last month, did you have conflict with your partner? (By conflict I mean verbal abuse, serious argument or violence, not a routine difference of opinion)* Women who answered: *Sometimes*, *Often* or *Always*—Ongoing domestic violence need identified *Never*—No ongoing domestic violence need identified, allocated to the comparison group Did not answer question (9.0%)—allocated to the comparison group
Child-custody	Women who answered Yes to *Do you have children*? AND Parenting issues identified on the re-entry treatment plan *Yes, issues identified*—included in the analysis *No issues identified*—allocated to the comparison group Women who reported not having children (22%) were excluded from the analysis	Q1. *Since release, was a child restored to your care?* Or Q2. *Since release, was a child removed from your care?* Women who answered *No* to Q1 or *Yes* to Q2—Ongoing child-custody need identified Women who answered *Yes* to Q1 or *No* to Q2—No ongoing child-custody need identified, allocated to the comparison group Did not answer question (75.5%)—allocated to the comparison group
Social support	Social support issues identified on the re-entry treatment plan *Yes, issues identified*—included in the analysis *No issues identified*—allocated to the comparison group	Q1. *Have you had any difficulties keeping in contact with your family or friends since your release?* Or Q2. *How often in the last month, did you have conflict with your relatives?* Women who answered *Yes* to Q1, or *Often* or *Always* to Q2—Ongoing social support need identified Women who answered *No, NA* or *Refused to answer* to Q1, or *Sometimes, Never* to Q2—No ongoing social support need identified, allocated to the comparison group Did not answer question (9.0%)—allocated to the comparison group
*Complex health issues*^*a*^		
Substance-use	Drug and/or alcohol issues identified on the re-entry treatment plan *Yes, issues identified*—included in the analysis *No issues identified*—allocated to the comparison group	Q1. *Have you used any drugs (other than prescribed) since your release?* Or Q2. *Have you had any problems with alcohol since release?* *Yes* to Q1 or Q2—Substance-use since release reported Women who answered *No* to Q1 or Q2, allocated to the comparison group Did not answer question (8.7%)—allocated to the comparison group
Mental Health	Women who received a mean score < 50 on the SF-12 mental component—included in the analysis Women who received a mean score > 50 on the SF-12 mental component—allocated to the comparison group Missing (9.2%)—allocated to the comparison group	Women who received a mean score < 50 on the SF-12 mental component—included in the analysis Women who received a mean score > 50 on the SF-12 mental component—allocated to the comparison group Missing (13.3%)—allocated to the comparison group

^a^ Complex health issues are measured at both baseline and follow-up; however, they are not computed as ongoing needs. Instead we analysed all patients who reported a health issue in the community regardless of whether they had an issue identified at baseline due to their harms on health, social functioning and associations with recidivism

6,

**Table 7:** Mediation of the effect of social determinants of health on substance-use through mental health

Relationship ^a^	Total effect	Direct effect (SE)	*p*-value	Indirect effect (SE)	Confidence intervals ^b^		Conclusion
					Lower bound	Upper bound	
Housing—> Mental health—> Substance-use	− 0.21	0.12 (0.26)	0.64	− 0.33 (0.10)	**0.16**	**0.57**	Full mediation
Employment—> Mental health—> Substance-use	− 0.22	− 0.32 (0.46)	0.48	0.10 (0.13)	− 0.13	0.36	No mediation
Financial—> Mental health—> Substance-use	0.51	0.37 (0.24)	0.12	0.15 (0.07)	**0.03**	**0.30**	Full mediation
Education—> Mental health—> Substance-use	0.71	− 0.19 (0.50)	0.70	0.90 (0.14)	− 0.17	0.38	No mediation
Domestic violence—> Mental health—> Substance-use	1.81	1.24 (0.63)	**0.05**	0.57 (0.23)	**0.21**	**1.10**	Partial mediation
Child-custody—> Mental health—> Substance-use	0.54	0.36 (0.30)	0.24	0.18 (0.10)	**0.01**	**0.39**	Full mediation
Social support—> Mental health—> Substance-use	0.84	0.48 (0.27)	0.07	0.36 (0.11)	**0.18**	**0.59**	Full mediation

Bold: Significance was determined if the entire interval lies on one side of the null hypothesis value (i.e., 0). This indicates statistical significance at the chosen significance level (i.e., 95% confidence level)

SE = standard error

^a^ Adjusted for admission year and age

^b^10,000 bootstrap samples were used for percentile confidence intervals

7.

## Abbreviations

SDOH: Social determinants of health
NSW: New South Wales
CSWs: Connections clinical support workers
RTC: Return-to-custody
No-RTC: Did not return-to-custody
SF-12: Short form-12 health survey
SPSS: IBM statistical package for the social sciences
AOR: Adjusted odds ratio
CI: Confidence interval
b: Beta efficiency
SE: Standard error
HREC: Human Research Ethics Committee
